# Effect of Laser Rescanning on the Characteristics and Residual Stress of Selective Laser Melted Titanium Ti6Al4V Alloy

**DOI:** 10.3390/ma13183940

**Published:** 2020-09-06

**Authors:** Xiaojin Miao, Meiping Wu, Jitai Han, Haohao Li, Xiu Ye

**Affiliations:** School of Mechanical Engineering, Jiangnan University, Wuxi 214122, China; 8202006305@jiangnan.edu.cn (X.M.); hanjitai@hotmail.com (J.H.); haohaoli163@163.com (H.L.); 15061883054@163.com (X.Y.)

**Keywords:** titanium Ti6Al4V alloy, SLM, rescanning, remelting, residual stress, mechanical properties

## Abstract

The titanium Ti6Al4V alloy has excellent properties, and is one of the most important and widely used metal materials in the field of modern high-tech. Selective laser melting (SLM) is an ideal process for the rapid prototyping of Ti6Al4V alloy components with complex structures, but the performances need to be further improved. In this paper, the relative density, hardness, and microstructure under different scanning conditions were first analyzed in order to clarify the role of rescanning process in improving the performances. Then, the effects of different scanning strategies on the residual stress were analyzed. The results show that the strategy of partition rescanning has the most significant effect on residual stress. Finally, the SLM experiments of aviation nozzle rings were carried out. The results show that the average residual stress of the Re-SLMed sample was reduced from 322 MPa to 254 MPa.

## 1. Introduction

The titanium Ti6Al4V alloy is a light alloy characterized by excellent mechanical properties and corrosion resistance, combined with low specific weight and potential biocompatibility [1], which is widely used in aerospace, chemical, and biomedical industries [2]. With the rapid development of modern science and technology, higher requirements are put forward for the structure and properties of titanium alloys. However, the ability of conventional technology to manufacture titanium alloy parts with a complex structure is very limited, and the cost is high, which makes it more and more difficult to meet the development needs of modern science and technology [3]. Selective laser melting (SLM) is an ideal process for the rapid prototyping of titanium alloy structural parts because of its high efficiency, low cost, high integration, and strong adaptability for complex structural parts [4,5]. However, the titanium alloy parts manufactured by SLM often have defects such as pores and cracks, which affect the mechanical properties. In addition, SLM is accompanied by frequent heating and cooling cycles, resulting in an excessive temperature gradient inside the part. The existence of a temperature gradient makes the thermal deformation of metal uneven, forming residual stress [6,7,8,9]. The existence of residual stress leads to deformation and cracks [10]. These problems greatly restrict the further development and application of SLMs.

Research shows that powder bed preheating is an effective method to reduce the residual stress [11,12,13], but a high preheating temperature is difficult to achieve in most commercial SLM machines [14]. Rescanning has been introduced as a new method to improve the performance of SLM in recent years. It is used for remelting the solidified layer during SLM. Remelting will change the temperature gradient, which will have a great effect on the relative density, surface roughness, microstructure, mechanical properties, and residual stress. Qiu et al. [15] rescanned the stainless steel 316L and found that the laser power plays a leading role in pore formation. The higher the rescanning laser power, the higher the relative density of 316L. Han et al. [16] studied the effect of laser remelting on the SLMed AlSi10Mg. They found that remelting helped to improve the surface quality of the samples. The surface roughness was up to 0.93 μm and the hardness increased by 19.5%. Demir et al. [17] proposed three different correction methods of laser remelting of the defected layer employing different scan strategies. The results indicated that these strategies could improve the density and surface quality effectively. Yasa et al. [18] found that laser remelting is a promising method to enhance the density and surface quality of SLM parts, at a cost of longer production times. The microhardness is improved in the laser remolten zone if sufficiently high-energy densities are provided. Wei et al. [19] thought that laser remelting is often used during SLM to improve the densification degree and top surface quality of the products, but research regarding its effects on residual stress, microstructure, and mechanical properties is still lacking. Liu et al. [20] also did a study of laser remelting the SLMed AlSi10Mg. They gained insight into the variation of the microstructures and microhardness, as well as the surface morphology, characteristics of molten pool, relative density, and phase identification. The results show that the remelting strategy in SLM improves the surface quality and relative density. Griffiths et al. [21] found that because of the reduced laser energy absorption and increased heat losses in the solid, the laser rescanning reduced the amount of columnar grains and increased the number of equiaxed grains. Xiao et al. [22] studied the effect of the rescanning cycle on the relative density, microstructure, residual stress, and mechanical properties of SLMed Ti6Al4V. The results show that the number of rescanning cycles has little effect on the microstructure. The relative density was the highest when the number of rescanning cycles is 1, while the ultimate tensile strength, yield strength, micro-hardness, and micro-strain are the highest when the number of rescanning cycle was 3. Haider et al. [23] pointed out that in the process of selective laser melting of a Ti6Al4V alloy, large residual stress is often produced because of a large temperature gradient, and the laser rescanning process can effectively reduce the residual stress.

Through pre-research, it was found that rescanning strategies have great effects on residual stress, which has not been studied in detail by experts. In this study, the performance of the selective laser remelted Ti6Al4V alloy was first analyzed by experiments in order to clarify the effect of the rescanning process on improving the performance. On this basis, several rescanning strategies were proposed, and the effects of rescanning strategies on residual stress were analyzed by simulation in order to select the best rescanning strategy. The research in this paper can guide the application of the rescanning process and provide ideas for improving the accuracy of SLM.

## 2. Materials and Methods

**Figure 1:**
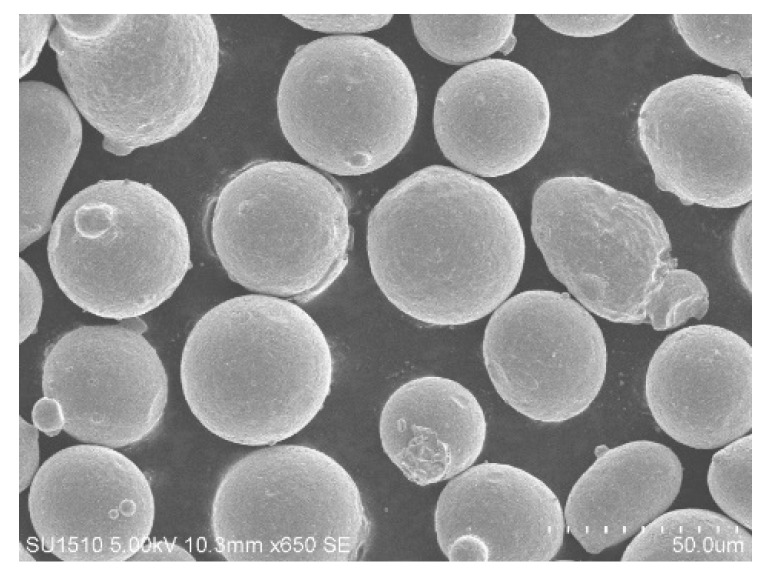
Titanium Ti6Al4V alloy powders.

In this paper, the titanium Ti6Al4V alloy was studied. Ti6Al4V, which is known as the “ace alloy”, has replaced the aluminum alloy and magnesium alloy in order to become the main material of important components of aero-engines. The Ti6Al4V powder used in the experiment has a good sphericity, as shown in Figure 1. The chemical composition of the powder is shown in Table 1.

In this paper, the relative density, hardness, and microstructure of the selective laser melted Ti6Al4V alloy samples with the rescanning process were first studied by experiments. By analyzing the performance of the Re-SLMed Ti6Al4V, the improvement effect of the rescanning process on the performance was clarified. Then, the effect of different rescanning strategies on residual stress was analyzed.

The samples were prepared using NCL-M2120 selective laser melting equipment (CHAMLION, Nanjing, China). The prepared samples were removed from the substrates by Wire cut Electrical Discharge Machining (WEDM). After cooling, the samples were ground and polished. The surface morphology and pore distribution of samples were observed with a LEICA-DM-2700M optical microscope (Leica, Heerbrugg, Switzerland). In this paper, the relative densities of the samples were calculated using the method of ImagePro. In the longitudinal section, three non-coincident regions were selected to calculate the relative density, individually, and the average value of the three was taken as the relative density of the sample. The Vickers-hardness of the sample was measured with an HVS-1000ZCM-XY digital microhardness tester (Suoyan Testing Instrument, Shanghai, China), taking a measuring point every 0.3 mm along the building direction. The applied load was 300 g and the holding time was 15 s. The hardness of all of the points was measured and the average value was taken as the hardness of the sample. The etchant reagent (HF:HNO_3_:H_2_O = 2:1:15) was prepared, and the cross-section of the sample after grinding and polishing was etched for about 15 s. Then, the microstructures of the samples were observed with a LEICA-DM-2700M optical microscope and Sigma300 scanning electron microscope (Carl Zeiss, Oberkochen, German, EHT = 20.00 kV, WD = 7.4 mm, Mag = 1.00 KX, and Signal A = SE2).

**Figure 2:**
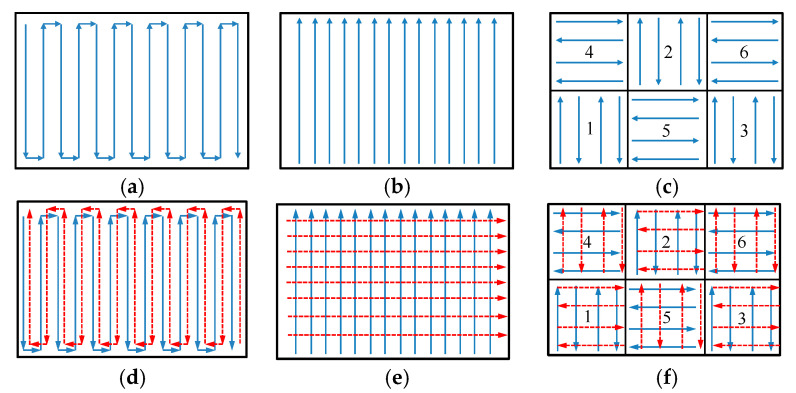
Scanning strategies: (**a**) S-shaped scanning; (**b**) one-way scanning; (**c**) partition scanning; (**d**) S-shaped rescanning; (**e**) one-way rescanning; (**f**) partition rescanning.

In this paper, the equivalent stresses of the SLMed and Re-SLMed samples were analyzed by simulation. It was found that the scanning strategies had a great effect on the residual stress [24,25]. Therefore, three different scanning strategies were used to study the stress distribution. The commonly used scanning strategies included S-shaped scanning, one-way scanning, and partition scanning. Different from ordinary scanning, this paper pays more attention to the rescanning. In order to optimize the stress, the strategies used in the second scanning were somewhat different from that in the first scanning. Figure 2 provided three different rescanning strategies. The blue solid line represents the path of the first scan, and the red dotted line represents the path of the second scan. As shown in Figure 2, the direction of the S-shaped rescanning strategy was opposite to that of the S-shaped scanning. The direction of the one-way rescanning strategy was rotated by 90° compared with that of the one-way scanning, and so was the partition rescanning strategy. The numbers on the partition represent the order of scanning.

The building of the simulation model was based on the Element Birth and Death of ANSYS. During the process of SLM, the temperature changed first, and the stress was mainly affected by the temperature distribution, which was in accords with the condition of indirect coupling. Therefore, indirect coupling was adopted in the simulation. The temperature field was simulated, and then the stress was analyzed by reading the temperature field. The solutions of the temperature field were applied to the stress field as load, and the solver type was transformed from temperature to stress. The size of the sample was 4.8 mm × 3.2 mm × 0.8 mm, and the size of the substrate was 6 mm × 4.5 mm × 1.6 mm. The bottom surface of the substrate was fully constrained. Mapped mesh was used in the model. The heat source model was a Gaussian heat source. Furthermore, the thermal convection, heat conduction, and thermal radiation were considered. In order to reduce the calculation time and improve the simulation accuracy, some reasonable premises and necessary instructions were made. (1) The powder bed was a continuous, homogeneous, and isotropic medium, which could be distinguished only by the thermophysical properties of the material. (2) The effect of powder shrinkage during melting was not considered. (3) The molten pool was even and smooth without vaporization and a capillary effect. The movement of the heat source was realized in the form of cyclic application and deletion of the thermal loads.

In order to reduce the influence of heat on the simulation accuracy, enthalpy was used to correct the latent heat. That is

(1) H=∫ρc(T)dT

where H is the enthalpy, ρ is the density of the material, and c(T) is the specific heat capacity.

The thermophysical properties of the powder and solid Ti6Al4V were considered using the calibrated values from Parry et al. [26], as shown in Table 2. The thermophysical properties of liquid Ti6Al4V refer to the research results of Bovineau et al. [27]. The melting point of Ti6Al4V was extracted as the upper limit of the scale in the simulation.

The setting of the process parameters is shown in Table 3. The parameters of the experiment and simulation were the same, and the parameters of Re-SLM and SLM are the same.

## 3. Results and Discussion

### 3.1. Relative Density and Hardness

**Figure 3:**
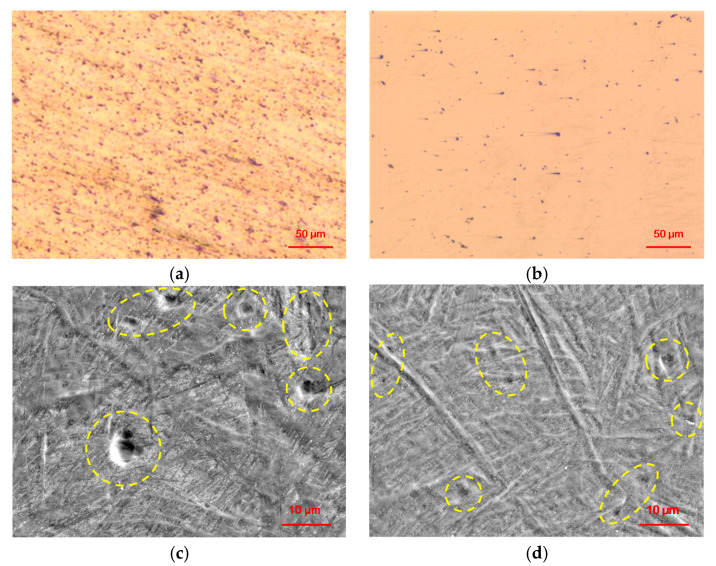
OM and SEM images of the Ti6Al4V samples under different scanning conditions: (**a**) OM image of the SLMed sample; (**b**) OM image of the Re-SLMed sample; (**c**) SEM image of the SLMed sample; (**d**) SEM image of the Re-SLMed sample.

The morphologies of the Ti6Al4V samples under different scanning conditions are shown in Figure 3. Figure 3a,b shows the optical microscope (OM) images of the SLMed and Re-SLMed samples, while Figure 3c,d shows the SEM images of the SLMed and Re-SLMed samples. The SEM images had a higher magnification, and the outline of the pores can clearly be seen. By comparing Figure 3a,b, it can be found that there are many pores on the SLMed Ti6Al4V, and the number of pores is much more than that on the Re-SLMed Ti6Al4V. The diameter and depth of the pores on the SLMed Ti6Al4V are large. They have a diameter of several microns, which is surrounded by many small pores, as shown in Figure 3c. In contrast, the diameter and depth of the pores on the Re-SLMed Ti6Al4V are much smaller, as shown in Figure 3d. The relative density of the remelted sample was greatly improved. It can be seen that the rescanning process had a positive effect on the relative density of the Ti6Al4V samples.

**Figure 4:**
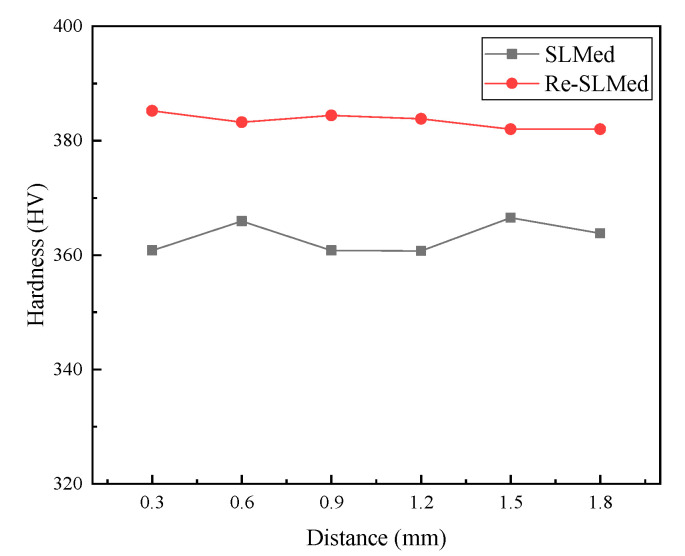
Hardness of the Ti6Al4V samples under different scanning conditions.

Figure 4 shows the hardness of the different samples at different points. It can be seen from the figure that the average hardness of the SLMed Ti6Al4V sample was slightly higher than 360 HV, which is similar to that of the Ti6Al4V formed by conventional technology. The high cooling rate of SLM could refine the grain and form an acicular martensitic α’ phase with a large aspect ratio, which could improve the hardness. However, the existence of pores reduced the hardness to a certain extent. Therefore, the hardness of SLMed Ti6Al4V was similar to that of Ti6Al4V formed by conventional technology. However, the hardness of Re-SLMed Ti6Al4V was more than 380 HV, which is about 5.5% higher than that of SLMed Ti6Al4V. This is mainly owing to the improvement of the relative density by remelting.

### 3.2. Microstructure

**Figure 5:**
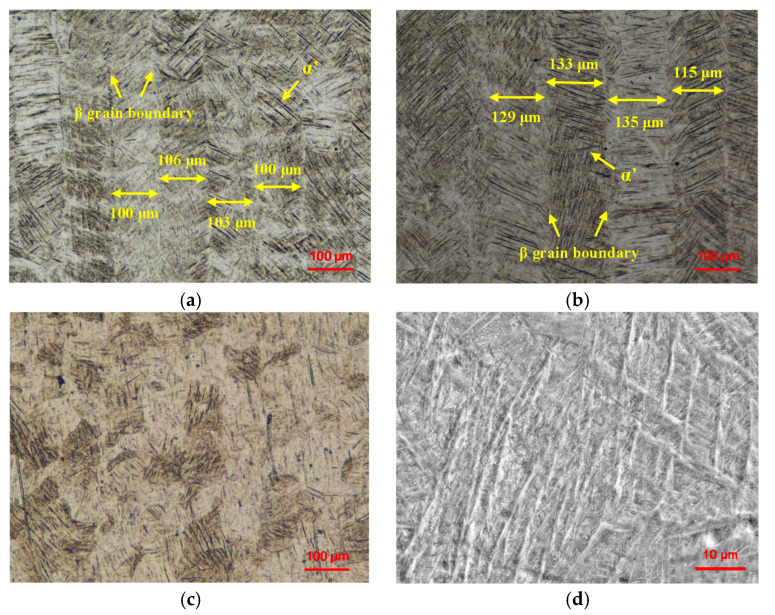
OM and SEM images of the microstructures of the Ti6Al4V samples: (**a**) OM image of the side view of SLMed Ti6Al4V; (**b**) OM image of the side view of Re-SLMed Ti6Al4V; (**c**) OM image of the top view of SLMed Ti6Al4V; (**d**) SEM image of α’ phase.

The microstructures of the samples under different scanning conditions are shown in Figure 5. Figure 5a,b are the side views. It can be seen from the figures that the β phase grows along the building direction in the form of a columnar crystal. The top view shows an equiaxed crystal rather than a columnar crystal, as shown in Figure 5c. Ti6Al4V is an alloy of α + β, and the acicular α’ phase is densely wrapped in the β phase. The grain boundary width of the β phase in the SLMed sample is slightly higher than 100 μm, while that of the remelted sample is extended to 130 μm. It can be seen that the width of the β grain boundary is extended by remelting.

During the first melting, the powders absorbed the energy and were rapidly heated to above the β phase transient temperature under the effect of a high-energy laser. This preferentially formed the β phase, and then formed a non-diffusion shear α’ phase during the subsequent rapid cooling [28]. Martensitic transformation occured along the β grain boundary to form a complete acicular martensitic α’ phase. With the laser moving, the molten pool cooled rapidly, and the cooling rate was greater than the critical cooling rate of 410 K/s, which is the correct rate for martensitic α’ formation [29]. The grain boundary of coarse β phase was obvious. In the process of laser remelting, the temperature of the molten pool rose again above the transient temperature of the β phase, and the acicular α’ phase decomposed. The acicular martensitic α’ phase was formed again during cooling, as shown in Figure 5d. Remelting did not eliminate the β grain boundary, but the grain boundary expanded.

### 3.3. Residual Stress

**Figure 6:**
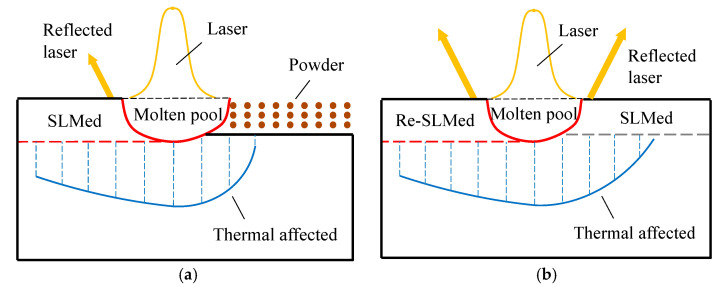
Processes of melting and remelting: (**a**) melting; (**b**) remelting.

Selective laser melting of metals is a complex thermodynamic process. When the laser has effects on the powders, part of the laser energy is reflected, and the rest is input into the powders. The reflection of powders to laser was weak, so the effective laser energy was high, and the width and depth of the molten pool were large, as shown in Figure 6a. However, during remelting, the target of the laser was the SLMed solid metal. The reflectivity of the solid metal to laser was much greater than that of the powder, so the laser absorption efficiency of the solid metal was low. The heat absorbed by the melted part was lower than that of the first melting, so the size of the molten pool of remelting was smaller than that of the first melting, as shown in Figure 6b. However, the thermal conductivity of the powder was weaker than that of the SLMed solid metal [30], so the effect of remelting on the sample was much greater, which had a positive effect on the stress distribution.

**Figure 7:**
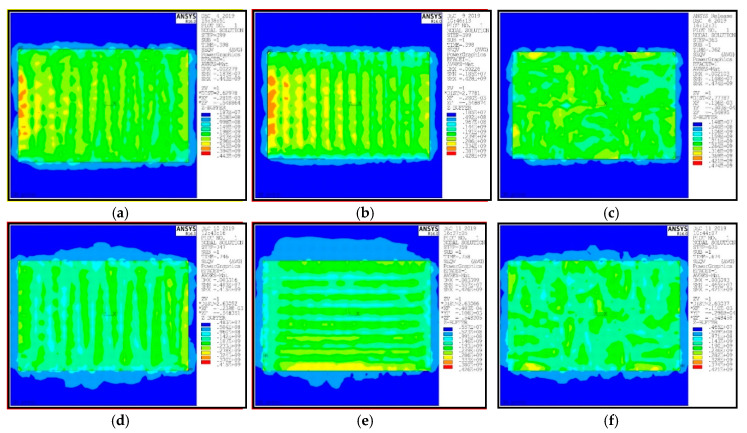
Von-Mises equivalent stress of the samples under different scanning conditions: (**a**) S-shaped scanning; (**b**) one-way scanning; (**c**) partition scanning; (**d**) S-shaped rescanning; (**e**) one-way rescanning; (**f**) partition rescanning.

The stress distributions of SLM and Re-SLM under different scanning strategies are shown in Figure 7. Figure 7a shows the von-Mises equivalent stress under the strategy of S-shaped scanning. It can be seen from the figure that the maximum equivalent stress of the sample is 443 MPa, which is located in the junction between the sample and the substrate. The equivalent stress at the center is about 296 MPa, and the principal stress on the upper surface of the sample is about 198 MPa. The temperature in the center of the molten pool is the highest. It decreased from the center to the surroundings during cooling. The closer to the center of the molten pool, the greater the temperature gradient and the greater the residual stress.

**Figure 8:**
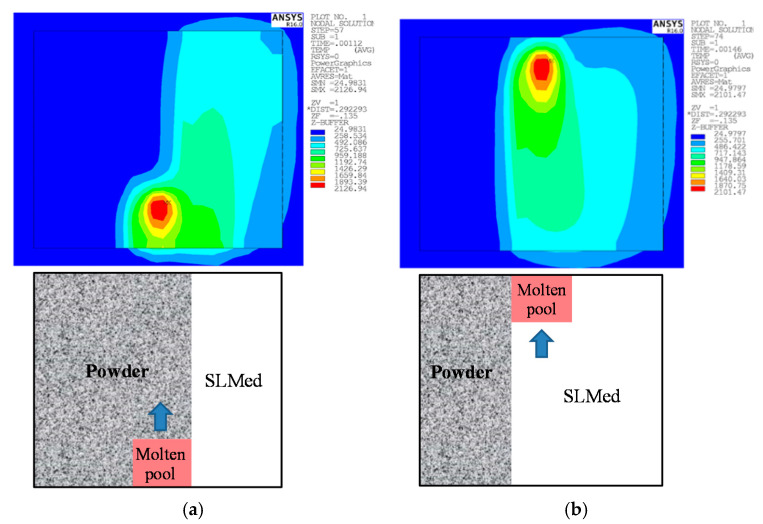
The molten pool in a melting path: (**a**) beginning; (**b**) end.

The residual stress under the strategy of S-shaped scanning decreased gradually from left to right in each melting path. At the beginning of SLM, the difference in temperature around the melting path was large. The non-melting part was affected by heat, and the temperature increased. The early stage of SLM had a preheating effect for the non-melting part, and the effect was more significant with time going on. Therefore, the temperature gradient in the later stage of SLM was small, which means that the residual stress was small. The range of the high stress zone at the beginning of each melting path was large, but was small at the end. The powders at the beginning of each melting path came into contact with the laser first. At this time, the material distributed around the molten pool was mainly powders, as shown in Figure 8. The low thermal conductivity of the powders led to the temperature concentration around the molten pool. This formed a large temperature gradient, and resulted in large residual stress. With the progress of SLM, the melted part solidified and the molten pool was mainly surrounded by SLMed solid Ti6Al4V. However, the thermal conductivity of the solid metal was high, and the temperature concentration around the molten pool was lower. The temperature gradient was small, so the range of the high stress zone was smaller.

**Figure 9:**
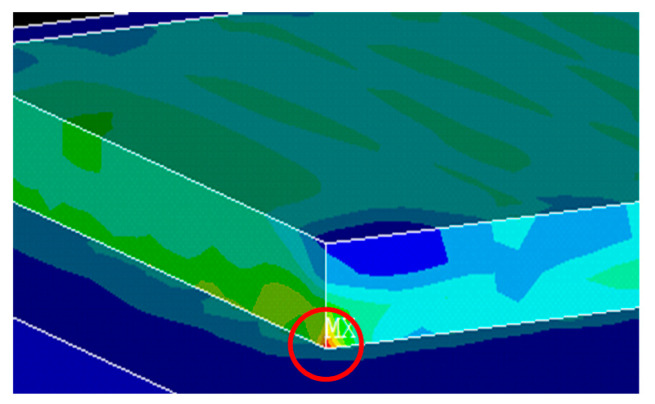
Stress concentration at the corner.

In addition, the substrate also restrained the thermal deformation of the sample. The substrate had a large heat dissipation area and relatively low temperature, so the temperature gradient at the joint of sample and substrate was large. It was easy to form a stress concentration at the corner of the sample, resulting in warpage, as shown in Figure 9.

The equivalent stress under the strategy of one-way scanning is shown in Figure 7b. It can be seen from the figure that the maximum equivalent stress of the sample is 428 MPa and the equivalent stress at the center is about 296 MPa, which are slightly smaller than that under the strategy of S-shaped scanning. The stress distribution under the strategy of one-way scanning was similar to that under the strategy of S-shaped scanning. The range of the high stress zone at the beginning of each melting path was large, and the stress decreased from left to right in each melting path. Figure 7c showed the equivalent stress under the strategy of partition scanning. This strategy was actually an optimized strategy of the strategy of S-shaped scanning. The sample was divided into several zones for different forms of S-shaped scanning. It can be seen that the equivalent stress distribution was better than the other two scanning strategies. The maximum equivalent stress of the sample was 414 MPa and the equivalent stress at the center was about 200 MPa. The high stress zones were mainly located at the junction of different partitions. The laser scanning path of each partition was perpendicular to that of its adjacent partition, so the principal stress directions were also perpendicular to each other. Therefore, the stress between the different partitions relaxed each other.

In order to improve the stress distribution, the scanning direction was adjusted during the second scan. The equivalent stress under the strategy of S-shaped rescanning is shown in Figure 7d. The residual stress of the Re-SLMed sample was significantly improved compared with the sample under the strategy of S-shaped scanning. In the second melting, the thermal effect of the laser was not as good as that of the first melting, and the heat dissipation of the solid Ti6Al4V was good, so the temperature gradient around the molten pool was low. The thermal effect caused by the molten pool was alleviated, and the thermal stress produced by the first scanning was reduced. Figure 7e shows the equivalent stress under the strategy of one-way rescanning. The stress distribution was similar to that under the strategy of one-way scanning, but the direction was different. Because of the change of direction of the second melting, the direction of the stress distribution also changed. On the whole, the stress improved a lot. The equivalent stress under the strategy of partition rescanning is shown in Figure 7f. The temperature gradient around the molten pool was small during the rescanning, which weakened the stress concentration at the junction of the adjacent partitions. The maximum residual stress decreased and the residual stress was well-distributed.

**Figure 10:**
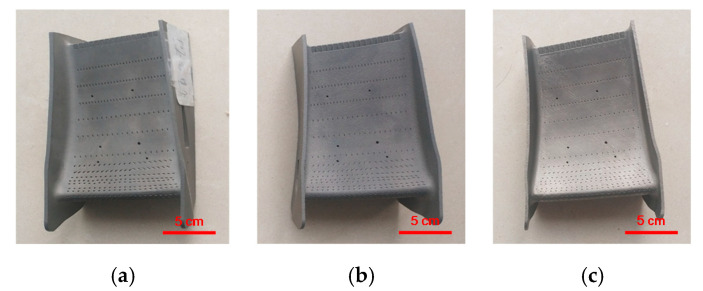
Melted aviation nozzle rings (**a**) SLMed; (**b**) Re-SLMed; (**c**) Re-SLM with parameters optimized

A group of experiments on selective laser melting of the aviation nozzle ring were carried out to verify the optimization effect of the rescanning process on the residual stress. As an important component of aero-engines, the working environment of the nozzle ring was harsh, and the requirement of internal residual stress was high. In addition to the scanning strategy, the process parameters also affected the melting quality. So, the experiment included three groups. The first group adopted the strategy of partition scanning. The second group adopted the strategy of partition rescanning, and the process parameters were consistent with the first group. The third group also adopted the strategy of partition rescanning, but the process parameters were optimized by regression analysis. The setting of parameters is shown in Table 4 and the melted samples are shown in Figure 10.

**Figure 11:**
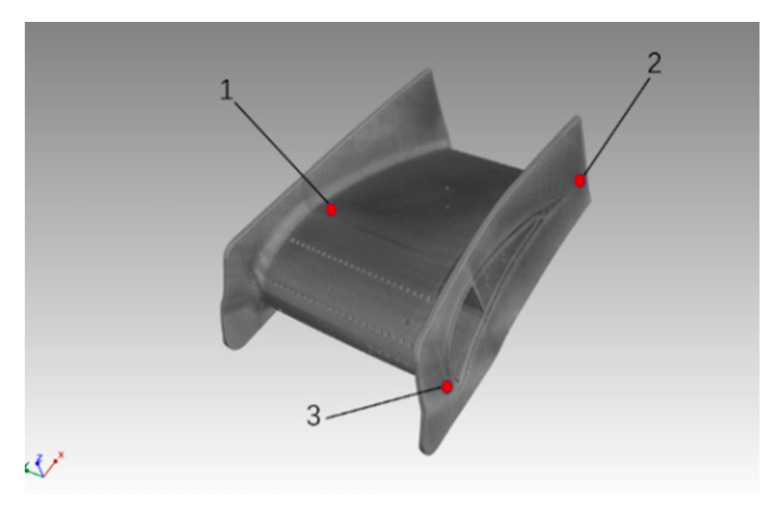
Residual stress measurement points.

**Figure 12:**
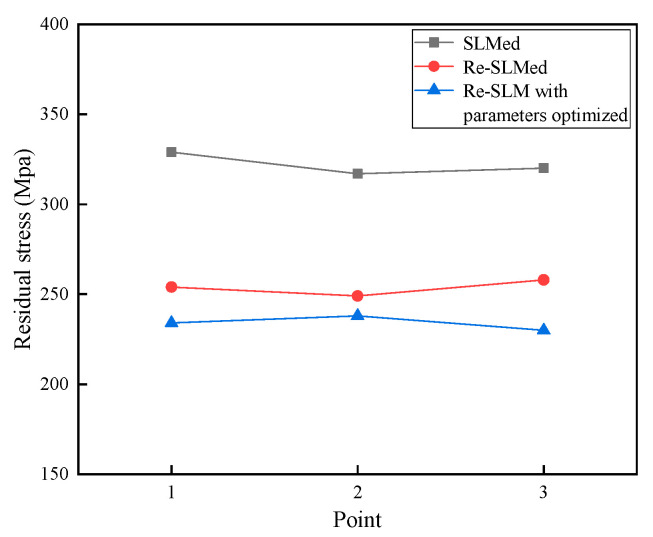
Residual stresses of the aviation nozzle rings.

Three points on each aviation nozzle ring were selected as the residual stress measurement points, as shown in Figure 11. The residual stresses of the aviation nozzle rings at the three points were determined by X-ray diffractometry, as shown in Figure 12. The average residual stresses at three points of each sample were 322 MPa, 254 MPa, and 234 MPa, respectively. It can be seen that the residual stress of the Re-SLMed sample was greatly improved compared with that of the SLMed sample. Although the parameter optimization could also improve the residual stress, the improvement was not significant.

**Figure 13:**
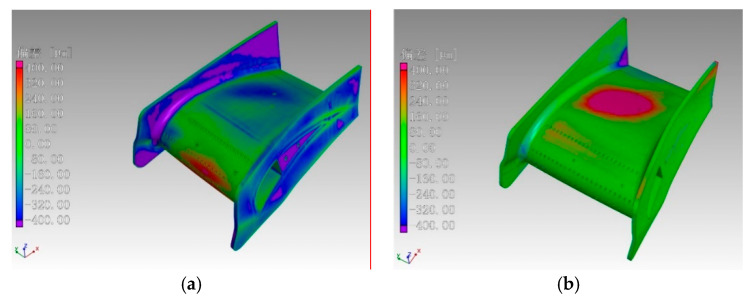
Dimensional deviation: (**a**) SLMed; (**b**) Re-SLMed.

The effect of the rescanning process on the forming accuracy was demonstrated by Yxlon FF35 industrial computerized tomography of the aviation nozzle rings. The models obtained by industrial computerized tomography were compared with the original 3D model in the software of Vgstudio MAX, as shown in Figure 13. It can be seen from the figure that the deviation of the SLMed sample was large, while that of Re-SLMed sample was much smaller.

## 4. Conclusions

In this paper, the rescanning process of the selective laser melting of the titanium Ti6Al4V alloy was studied. The relative density, hardness, and microstructure of Ti6Al4V samples under different scanning conditions were compared and analyzed. In addition, the effects of rescanning strategies on residual stress were studied. It is considered that the rescanning process is helpful to improve the selective laser melting properties, optimize the residual stress, and reduce the size deviation of the Ti6Al4V alloy.

1. The rescanning process could improve the relative density of the Ti6Al4V alloy. The number and size of the pores were greatly reduced. Because of the improvement in the relative density, the hardness was also improved. As for the microstructure, the grain boundary of the β phase was greatly expanded by remelting.
2. Different scanning strategies had different effects on residual stress distribution. The strategy of partition scanning had the best residual stress distribution in SLM. The size and distribution of residual stress could be effectively improved by the process of rescanning. In contrast, the strategy of partition rescanning had the most significant effect on the improvement of residual stress.
3. The optimization of the process parameters could also improve the residual stress, but the improvement was not as good as the rescanning process. By optimizing the residual stress, the rescanning process was helpful at reducing the deviation caused by excessive residual stress.
4. The rescanning parameters studied in this paper were consistent with those of the first scanning. However, if the parameters of the two scanning were inconsistent, the improvement could be different. Further research is needed on this point.

## Figures and Tables

**Table 1:** Chemical composition of the titanium Ti6Al4V alloy powder.

Chemical Composition	Al	Fe	V	C	O	N	H	Ti
**Proportion (wt.%)**	6.0–6.75	≤0.02	3.5–4.5	≤0.02	≤0.09	≤0.01	≤0.002	Bal.

**Table 2:** Thermophysical properties of Ti6Al4V.

Temperature (°C)		Specific Heat Capacity (J/kg·K)	Density (g/cm^3^)	Thermal Diffusivity (m^2^/s)	Thermal Conductivity (W/mK)
20	Powder	520	2.65	0.11	0.145
	Solid	543	4.42	2.95	7.07
200	Powder	505	2.64	0.08	0.104
	Solid	566	4.39	3.74	9.28
400	Powder	480	2.62	0.07	0.083
	Solid	599	4.36	4.51	11.8
600	Powder	472	2.60	0.14	0.167
	Solid	636	4.33	5.25	14.5
800	Powder	507	2.58	0.21	0.279
	Solid	675	4.30	5.98	17.4
1000	Powder	610	2.56	0.52	0.813
	Solid	713	4.27	7.22	22.0
1200	Powder	808	2.54	0.53	1.09
	Solid	745	4.24	7.91	25.0

**Table 3:** Setting of process parameters.

Process Parameters	Laser Power (W)	Scanning Speed (mm/s)	Layer Thickness (μm)	Hatch Spacing (mm)
Value	120	1200	30	0.075

**Table 4:** Setting of parameters.

No.		Laser Power (W)	Scanning Speed (mm/s)	Hatch Spacing (mm)
1	First Scanning	120	1200	0.075
	Second Scanning	-	-	-
2	First Scanning	120	1200	0.075
	Second Scanning	120	1200	0.075
3	First Scanning	120	1200	0.075
	Second Scanning	111	1454	0.096

## References

[1] Jesus J.S., Borrego L.P., Ferreira J.A.M., Costa J.D., Capela C. (2019). Fatigue crack growth behaviour in Ti6Al4V alloy specimens produced by selective laser melting. Int. J. Fract..

[2] Bilgin G.M., Esen Z., Akın Ş.K., Dericioğlu A.F. (2017). Optimization of the mechanical properties of Ti-6Al-4V alloy fabricated by selective laser melting using thermohydrogen processes. Mater. Sci. Eng. A.

[3] Leon A., Levy G.K., Ron T., Shirizly A., Aghion E. (2020). The effect of strain rate on stress corrosion performance of Ti6Al4V alloy produced by additive manufacturing process. J. Mater. Res. Technol..

[4] Yan X., Shi C., Liu T., Ye Y., Chang C., Ma W., Deng C., Yin S., Liao H., Liu M. (2020). Effect of heat treatment on the corrosion resistance behavior of selective laser melted Ti6Al4V ELI. Surf. Coatings Technol..

[5] Han J., Wu M., Duan W. (2020). A Proposed Scan Strategy Used on SLM Inner Structure Part. Materials.

[6] Mercelis P., Kruth J. (2006). Residual stresses in selective laser sintering and selective laser melting. Rapid Prototyp. J..

[7] Zaeh M.F., Branner G. (2009). Investigations on residual stresses and deformations in selective laser melting. Prod. Eng..

[8] Hodge N.E., Ferencz R.M., Vignes R. (2016). Experimental comparison of residual stresses for a thermomechanical model for the simulation of selective laser melting. Addit. Manuf..

[9] Mishurova T., Cabeza S., Thiede T., Nadammal N., Kromm A., Klaus M., Genzel C., Haberland C., Bruno G. (2018). The Influence of the Support Structure on Residual Stress and Distortion in SLM Inconel 718 Parts. Met. Mater. Trans. A.

[10] Shipley H., McDonnell D., Culleton M., Coull R., Lupoi R., O’Donnell G., Trimble D. (2018). Optimisation of process parameters to address fundamental challenges during selective laser melting of Ti-6Al-4V: A review. Int. J. Mach. Tools Manuf..

[11] Shiomi M., Osakada K., Nakamura K., Yamashita T., Abe F. (2004). Residual Stress within Metallic Model Made by Selective Laser Melting Process. CIRP Ann. Manuf. Technol..

[12] Le Roux S., Salem M., Hor A. (2018). Improvement of the bridge curvature method to assess residual stresses in selective laser melting. Addit. Manuf..

[13] Loh L.E., Chua C.K., Yeong W.Y., Song J., Mapar M., Sing S.L., Liu Z.-H., Zhang D.-Q. (2015). Numerical investigation and an effective modelling on the Selective Laser Melting (SLM) process with aluminium alloy 6061. Int. J. Heat Mass Transf..

[14] Ali H., Ma L., Ghadbeigi H., Mumtaz K. (2017). In-situ residual stress reduction, martensitic decomposition and mechanical properties enhancement through high temperature powder bed pre-heating of Selective Laser Melted Ti_6_Al_4_V. Mater. Sci. Eng. A.

[15] Qiu C., Wang Z., Aladawi A.S., Al Kindi M., Al Hatmi I., Chen H., Chen L. (2019). Influence of Laser Processing Strategy and Remelting on Surface Structure and Porosity Development during Selective Laser Melting of a Metallic Material. Met. Mater. Trans. A.

[16] Han Q., Jiao Y. (2019). Effect of heat treatment and laser surface remelting on AlSi_10_Mg alloy fabricated by selective laser melting. Int. J. Adv. Manuf. Technol..

[17] Demir A.G., Previtali B. (2017). Investigation of remelting and preheating in SLM of 18Ni300 maraging steel as corrective and preventive measures for porosity reduction. Int. J. Adv. Manuf. Technol..

[18] Yasa E., Deckers J., Kruth J.-P. (2011). The investigation of the influence of laser re-melting on density, surface quality and microstructure of selective laser melting parts. Rapid Prototyp. J..

[19] Wei K., Lv M., Zeng X., Xiao Z., Huang G., Liu M., Deng J. (2019). Effect of laser remelting on deposition quality, residual stress, microstructure, and mechanical property of selective laser melting processed Ti-5Al-2.5Sn alloy. Mater. Charact..

[20] Liu B., Li B.-Q., Li Z. (2019). Selective laser remelting of an additive layer manufacturing process on AlSi_10_Mg. Results Phys..

[21] Griffiths S., Rossell M.D., Croteau J., Vo N., Dunand D., Leinenbach C. (2018). Effect of laser rescanning on the grain microstructure of a selective laser melted Al-Mg-Zr alloy. Mater. Charact..

[22] Xiao Z., Chen C., Hu Z., Zhu H., Zeng X. (2020). Effect of rescanning cycles on the characteristics of selective laser melting of Ti_6_Al_4_V. Opt. Laser Technol..

[23] Ali H., Ghadbeigi H., Mumtaz K. (2018). Effect of scanning strategies on residual stress and mechanical properties of Selective Laser Melted Ti_6_Al_4_V. Mater. Sci. Eng. A.

[24] Carter L.N., Martin C., Withers P.J., Attallah M.M. (2014). The influence of the laser scan strategy on grain structure and cracking behaviour in SLM powder-bed fabricated nickel superalloy. J. Alloy. Compd..

[25] Salmi A., Atzeni E. (2017). History of residual stresses during the production phases of AlSi_10_Mg parts processed by powder bed additive manufacturing technology. Virtual Phys. Prototyp..

[26] Parry L., Ashcroft I., Wildman R. (2016). Understanding the effect of laser scan strategy on residual stress in selective laser melting through thermo-mechanical simulation. Addit. Manuf..

[27] Boivineau M., Cagran C., Doytier D., Eyraud V., Nadal M.-H., Wilthan B., Pottlacher G. (2006). Thermophysical Properties of Solid and Liquid Ti-6Al-4V (TA6V) Alloy. Int. J. Thermophys..

[28] Zhao Z., Li L., Bai P., Jin Y., Wu L., Li J., Guan R.-G., Qu H.-Q. (2018). The Heat Treatment Influence on the Microstructure and Hardness of TC4 Titanium Alloy Manufactured via Selective Laser Melting. Materials.

[29] Ahmed T., Rack H. (1998). Phase transformations during cooling in α+β titanium alloys. Mater. Sci. Eng. A.

[30] Chen C., Yin J., Zhu H., Xiao Z., Zhang L., Zeng X. (2019). Effect of overlap rate and pattern on residual stress in selective laser melting. Int. J. Mach. Tools Manuf..

